# Detailed protein sequence alignment based on Spectral Similarity Score (SSS)

**DOI:** 10.1186/1471-2105-6-105

**Published:** 2005-04-23

**Authors:** Kshitiz Gupta, Dina Thomas, SV Vidya, KV Venkatesh, S Ramakumar

**Affiliations:** 1 Department of Computer Science & Engineering, Indian Institute of Technology, Bombay, Mumbai, India; 2 Department of Chemical Engineering, Indian Institute of Technology, Bombay, Mumbai, India; 3 School of Biosciences & Bioengineering, Indian Institute of Technology, Bombay, Mumbai, India; 4 Department of Physics, Indian Institute of Science, Bangalore, India; 5 Bioinformatics Center, Indian Institute of Science, Bangalore, India

## Abstract

**Background:**

The chemical property and biological function of a protein is a direct consequence of its primary structure. Several algorithms have been developed which determine alignment and similarity of primary protein sequences. However, character based similarity cannot provide insight into the structural aspects of a protein. We present a method based on spectral similarity to compare subsequences of amino acids that behave similarly but are not aligned well by considering amino acids as mere characters. This approach finds a similarity score between sequences based on any given attribute, like hydrophobicity of amino acids, on the basis of spectral information after partial conversion to the frequency domain.

**Results:**

Distance matrices of various branches of the human kinome, that is the full complement of human kinases, were developed that matched the phylogenetic tree of the human kinome establishing the efficacy of the global alignment of the algorithm. PKCd and PKCe kinases share close biological properties and structural similarities but do not give high scores with character based alignments. Detailed comparison established close similarities between subsequences that do not have any significant character identity. We compared their known 3D structures to establish that the algorithm is able to pick subsequences that are not considered similar by character based matching algorithms but share structural similarities. Similarly many subsequences with low character identity were picked between xyna-theau and xyna-clotm F/10 xylanases. Comparison of 3D structures of the subsequences confirmed the claim of similarity in structure.

**Conclusion:**

An algorithm is developed which is inspired by successful application of spectral similarity applied to music sequences. The method captures subsequences that do not align by traditional character based alignment tools but give rise to similar secondary and tertiary structures. The Spectral Similarity Score (SSS) is an extension to the conventional similarity methods and results indicate that it holds a strong potential for analysis of various biological sequences and structural variations in proteins.

## Background

Comparison and alignment of primary structures has become the prime tool for protein sequence analysis [1]. Comparative analysis of primary structures of amino acids can reveal useful information regarding the structure and function of proteins. Many algorithms therefore have been developed and databases designed to search for similar proteins, but most of them are based on character-matching techniques. In this technique, the amino acids are considered to be distinct characters.

However, there are certain limitations of character based similarity measure approaches that cannot allow insights in the structural aspects of the protein. Though two sequences with a high character based similarity are expected to depict similar structures and show close biological functions, the reverse is not always true. Instances have been found where structurally closely related sequences do not provide good character based similarity measure [2]. Two protein sequences with low sequential identity may show similarities in their physiochemical properties, tertiary structure and biological activities. There could be many reasons for this observation. The one most widely hypothesized is that nature sometimes retains the biological functions but changes the amino acids as the protein evolves. Also, most of the times, researchers are interested in the active site of the protein, and not its overall backbone structure. The active site may occupy just a small part of the overall protein, therefore it is important to capture the structure and local variations in properties of amino acids at a certain location. Overall similarity score based on character matching may not be able to capture the local similarities, particularly if the amino acids differ in the location but provide similar overall structure.

Many algorithms have been developed based on character based similarity, though differing in their approaches. BLAST attempts to fragment protein sequences and establishes matches between them using substitution matrices for thresholds. PSI-BLAST [3], an extension to BLAST [4,5], uses similarity matrices (called profiles) based on specificity of position of an amino acid, and is probably the most widely used sequence similarity tool. All BLAST algorithms are based on consideration of sequences as long strings of alphabets. In addition, various heuristics are employed based on biological observations as extensions to purely character based approaches. Similarly, FASTA [6] algorithms using optimized gap penalties are used to find homologous sequences from protein databases. SSearch [7] engine implements Smith-Waterman [8] algorithm, an extension to the N-W algorithm [9] for establishment of protein similarity. PRIDE [10] establishes similarity score by considering *C*^*α *^- *C*^*α *^distances between residues separated within a threshold of amino acids. An interesting holistic approach to protein alignment developed by Taylor and Orengo [2] present an algorithm that considers structural aspects inducing hydrogen bonding like solvent exposure, torsion angle apart from the traditional character based methods, and does indeed presents appreciable alignments for proteins with low sequence similarity. Tonges *et al. *[11] presents a general method for sequence alignment based on conventional dynamic programming and building of secondary matrices by their results. However, it works best for highly homologous sequences and therefore is of not much use for less homologous sequences. Double dynamic programming approach, an interesting extension to the N-W [9] algorithm is used to increase the accuracy in multiple sequence alignment by Tailor *et al. *[12]. T-Coffee [13] also shows appreciable enhancement in accuracy over traditional alignment methods by prearchiving of alignment information. CHAIN [14] uses monte carlo optimization of a hidden markov model to establish gapped alignment of primary structures. A whole range of CLUSTAL [15,16] softwares are available for protein alignment customized for specific needs and available resources. Further, machine learning approaches [17] have been used to improve the similarity searches. Pearson [18] and Shpaer et al. [19] provide an extensive review and comparison of the existing tools for searching primary protein sequence databases. However, the algorithms fail to extract subsequences that are not identical in characters but share common secondary structure. In all of the above, similarity is very closely related to identity except while incorporating discrete properties like acidic, basic, aromatic to which an aligned amino acid may belong to.

Non character based approaches to establish similarity between polypeptides have also been tried with limited success like by capturing the repetitions of amino acids by considering sequences in the frequency domain using the acclaimed Fast Fourier Transformation [20-22]. Various *repeats *in the protein sequences can be adequately captured by using FFT and its various versions, but we lose the sequence information in such attempts.

Most of the algorithms for similarity detection are primarily alignment tools and are based on string managements of protein sequences that are considered as words of 20 characters. The algorithm presented here attempts to remove this limitation by considering the properties of the amino acids and also their variation directly during matching of sequences. Our approach is inspired by a few recent researches in the field of music retrieval and the commercial success of Music Database and Retrieval Systems [23] (MDR) based on the Spectral Analysis of audio signals. We have attempted to use the ideas in the field advantageously along with the traditional methods to adapt to protein sequence similarity estimation. Since the MDRs have been commercialized, new algorithms and heuristics may not be available in the public domain. The developed algorithm is capable of evaluating similarity based on any or a combination of the 256 attributes listed down in the AA index database [24,25] and is intended to detect *local variations *in the property in the sequence along with global alignment. We present this method as an extension to traditional character based matching algorithm.

## Results

The algorithm was coded, with *S*_*z *_and *F *kept as variable parameters. A single property, i.e. *hydrophobicity *[26] was taken as the property, *F *is kept more than twice the *S*_*z *_so that no information is lost while the neighborhood around the highest peak is considered. *β*_*p*_, the penalty factor can be changed to accommodate the parameters and can be tuned to consider the 'not so similar' segments in the sequences. The threshold for selection of subsequences of size 8 amino acids with *β *= 2.5, was kept as a function of the actual character identities in the subsequences. The threshold *t *was taken as *SSS *< = 3.5 - n * 0.4, so that if there is no character identity, subsequence matches with *SSS *< = 3.5 were looked for. This non-fixed threshold function was evolved as matchings with high character identity did produce low matches, but the "interesting" matches are typically the ones with low identity of amino acids. A detailed analysis of the matching presents subsequences that are alphabetically dissimilar, and are therefore not detected by traditional algorithms, but share common 3D structures.

1. Various branches of the evolutionary tree of Human Kinome [27] were generated by tree-generating algorithm, after finding the distance measure for various kinases. As an illustration, when closely related kinases (with SwissProt accession no. in brackets), *PAK4 (O96013), PAK5 (O95547), PAK6 (Q9NQU5) *and a distant neighbor *PLK1 *(SwissProt acc.no: P53350) are run through the automation of the algorithm, expected results are obtained (see table 2). This establishes the global alignment capability which is due to the Dynamic Programming Algorithm. Similarly evolutionary relationships were found for the *PKC *series of human kinases (see table 3) with *F *doubled. The global alignment capability does not seem to be dependent on the *F *measure significantly.

2. *PKCd *(pdbid [28,29] (accession number in the Protein Data Bank [28]): 1*bdy*) and *PKCe *(pdbid: 1*GMI*) (BLAST identity 40%, similarity 57%) human kinases are considered as evolutionarily similar but do not produce close alignments (GAP 55.472%, SSS .7149). The algorithm was able to identify many subsequences that are not identical but share close secondary structure similarity. Results are tabulated in table 4 alongwith the alignment found in BLAST. Also, results are compared with those of Smith-Waterman algorithm [8], using the standard software called SSearch [7]. In both the cases, it was seen that SSS was able to identify subsequences that are alphabetically dissimilar but gives low SSS scores, but are structurally similar. The value of segment size *S*_*z *_was kept 8 and *F *16. The tertiary structures of the subsequences within the threshold were found to be closely similar using Swiss pdbviewer (SPBDV) [30,31]. The references of the figure showing alignments are given in each row in table 4. The alignments shown is between the subsequences by a simple "Magic Fit" in SPDBV using the actual pdb files of the proteins, and most of the fits obtained for SSS within the threshold validate our results. Therefore, it is possible that even when the subsequences have complete identity, they may theoretically not fit at all in the actual protein owing to the non-alignment of other regions.

PKCd and PKCe, though share a similar fold, do not superimpose well using SPDBV but our experiments suggest that the subsequences picked up by SSS within the threshold do produce good fits with low rms (root mean square) value apart from their similarity in the secondary structure (also shown in the table 4). Figure 4 shows the fits obtained using SPDBV for subsequences that were picked by the algorithm with the exception of Figure 6 which reported a high SSS value, and also has reported a high rms value during pdb fitting. Matches found with high character identity are not shown in the table, but in general their SSS value is lower which is taken care of by the threshold. This demonstrates that the algorithm's ability to pick non identical subsequences if they are similar in their tertiary structure. The accounting of subsequences through SSS that are found below threshold would increase the BLAST similarity score by more than 10% in this particular example and more than 5% in most other protein pairs. However, the potency of the algorithm essentially remains in capturing "interesting" subsequences and not perse at global alignment.

3. SSS consistently was found to capture subsequences with similar secondary structures, and most of the times with similar tertiary structures purely by the primary structure. In *xyna-psefl *(pdbid: 1*clx*) and *xynz-clotm chain A *(pdbid: 1*xyz*) we found interestingly subsequences that do not get aligned in BLAST but still show similar tertiary structures using the algorithm. Table 6 shows subsequences that are not aligned in BLAST and do not share sequential similarity but are similar in tertiary structures as seen through their pdb coordinates. Similar conclusions can be drawn by comparison with the results obtained by Smith-Waterman algorithm. SSearch engine was used for comparative analysis. This strongly suggests the potency of the algorithm to even find non aligned subsequences that are structurally similar and renders SSS as a useful test after traditional alignment algorithms. This seems to be a result of the inadequacy of the simplistic dynamic programming approach compared to BLAST which is a better alignment tool, but depicts that SSS with better alignment tools as an abstraction (like the way dynamic programming is used as a wrapper) can be used effectively for finding alignments between proteins where homology is not detected using traditional algorithms.

4. The algorithm was run on *xyna-theau *(pdbid: 1*gor*) and *xynz-clotm chain A *(pdbid: 1*xyz*) and compared with the results from BLAST. Subsequences that were found to be matching with large distance values (meaning that the similarity is not very high, but reported in the matching segments) were looked for their secondary structures. Appreciable similarity in secondary structures were reported though alignment was not perfect (see table 5). Figures 5 shows the fits obtained for the individual subsequences picked by the SSS using SPDBV. Xyna-theau and xynz-clotm are abound in *H *(Helix), but the algorithm is able to catch the subsequences where for short duration *β *strands were located within two bends and align them with a similar stretch in the other sequence. It must be considered, that interesting results may be expected by the algorithm (and those not expected from character based alignment) only when the distance value *D *is not very small, and a micro analysis of the matching segments may produce results that are unobtainable otherwise.

5. *xyna-theau *(pdbid: 1*gor*) and *xyna-strli *(pdbid: 1*eov*) when run over by the algorithm also produced subsequences that were dissimilar in characters but highly similar in their overall structure. In Table 7, subsequences 3 and 4 were completely dissimilar sequences but were obtained by the algorithm and were found to be very similar in their tertiary structure with very low rms values. Both the subsequences produce *α *helical structures.

This illustrates the chief advantage of the algorithm, wherein not only direct character alignment but similarity between subsequences is captured. Analysis in the spectral domain after conversion to an orthogonal plane of property using FFT allows SSS to establish similarity where traditional character based algorithm may not succeed. This holds true for BLAST and many other algorithms based on a similar approach. Though, essentially SSS is suited for detailed analysis of sequences in a locality and can be wrapped over by other global alignment tools (like N-W dynamic programming or BLAST), but within the locality it scores over other algorithm due to its emphasis on the *local variation of the property *besides the property itself. As has been demonstrated in the results, local variation of a group of properties can also have an effect on determining the structural and functional properties of the protein in a locality. Therefore, it scores over even Smith-Waterman [8] in the cases where alphabetical similarity is either low or does not exist. Further, any purely character based similarity approach cannot capture the local variation of multiple properties in a local region. If two subsequences register an appreciably low SSS score, and are sequentially different, it depicts the local variation of property (here hydrophobic effect for residue burial) to be similar in both the subsequences, which might be of interest to the analyst. Taking a greater frequency component (*F *being doubled) and subsequent analysis at such locations might give useful insight into the similarity pattern where character matching is not evident. The flexibility to use the algorithm with a healthy compromise between the frequency and position offers another advantage of the developed algorithm. Further, other properties like *α *helical propensity, *β *strand propensity may be used in conjunction with hydrophobicity as different property planes.

## Conclusion

We present a novel method to establish similarity between two amino acid sequences that goes further than the conventional character based similarity approaches and purely frequency based similarity approaches based on repetitions of amino acids. The algorithm derives its inspiration from spectral similarity approaches employed successfully in music database retrieval systems and attempts to establish similarity based on the Spectral Similarity Score on any general attribute of amino acids. We have demonstrated that the approach is capable of picking subsequences of amino acids as similar though they may not be identical in nature. Further, tertiary structures of these picked subsequences have shown appreciable similarity and fit, though the overall structure of the protein may not fit well. This demonstrates that the algorithm is capable of establishing similarity in tertiary structure purely by processing primary structures even when the primary subsequences do not match well. Further, as SSS is able to find even subsequences that do not align through BLAST or SSsearch but are nevertheless similar, it can be used as a useful tool after operation by traditional alignment algorithms. Further, SSS without dynamic programming can be used to pick a subsequence of interest from a corpus of subsequences that alignment algorithms would fail to achieve.

A distinct advantage of the algorithm is its ability to detect subsequences that are not similar in characters but in the property under consideration, and even in the profile of the local variation of the property in a localized region. Therefore, it is able to establish similarity in those subsequences where character based similarity is not possible to establish. The algorithm is flexible and allows alteration of *size *of subsequences as powers of 2. If FFT is replaced by other fourier transformation algorithm (at the cost of time complexity) then this constraint on the size of the subsequence may also be eliminated. Another advantage of the algorithm is its ability to encode any property of the amino acids as given in the AAindex database. Therefore different indices may be used in different contexts to establish similarity in function, fold, structural, or evolutionary or superfamily relationships. These indices may be normalized to compare the results from different indices. Further, multiple properties may be handled at a time either by generating *property profiles *in different planes or by creating a new property as a linear combination of multiple properties. Effects of such extensions are currently being explored.

The Dynamic Programming approach can be replaced by other approaches used in character based similarity establishment with suitable modifications. Smith-Waterman algorithm performs an exhaustive search of all possible gapped alignments between a pair of sequences using a set of scoring parameters, and therefore can be used more effectively with SSS. It is noteworthy, that though there are frequency conversion mechanisms other than FFT, but the latter is a linear time algorithm and is therefore, faster. If Smith-Waterman algorithm is used as a wrapper for an exhaustive search of gapped alignments (here, SSS similarity alignments), usage of FFT would become critically important. Penalty, windowing and normalization parameters may be further tuned to get better depth in the results. Histograms can be generated for a better visualization of similarity and to avoid detailed analysis of the SSS results. Color coding of alignment, as done in BLAST, can be employed and algorithms used in MDR may be used in filtering and linearity enforcing. This approach, we believe, can be used in many fields in bioinformatics to establish similarity. This algorithm can be effectively used to find similarity in genomes after suitable estimation of the parameters, and can also be used to find similarities in the 3-dimensional structures of proteins by using variations in relatively accessible surface areas of proteins.

## Methods

The focus of the SSS algorithm is to capture subsequences in amino acid sequences that are not similar on alphabetical scale, but are similar on some property(s) scale of which a choice can be made during the course of the algorithm. SSS involves preprocessing of the primary structure, and conversion to the frequency domain followed by matching and estimation of the similarity score.

### Preprocessing of inputs

The algorithm intends to find the similarity measure based on any general attribute of the amino acid. Therefore, the amino acid in the input sequence is replaced with its attribute measure, such as the hydrophobicity [26] value. This generates a *property profile *of the protein in one dimension, which is a sequence of floating point numbers of length equal to the number of amino acids in the protein. If more than one property is to be considered simultaneously, then the *property profile *is a multi dimensional sequence.

Formally, for a protein *Pr *of size *n *(number of amino acids) let the *p *properties considered be {*P*_1_, *P*_2_,..., *P*_*p*_}. Let function *P*_*p*_(*n*) give the property value of type *P*_*p *_of the amino acid at position *n *in protein *Pr*. Then the *property profile *of *Pr *is designed as



**Figure 1:**
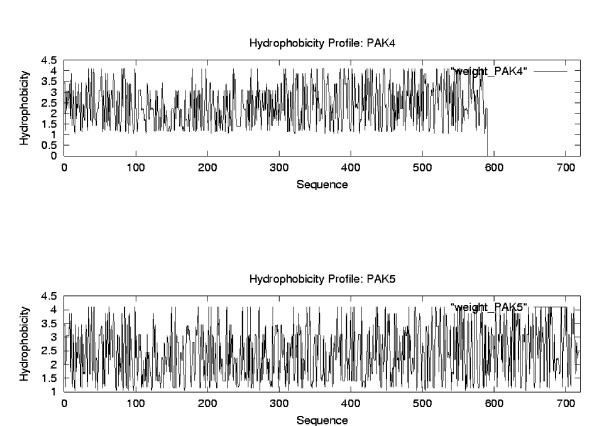
**Hydrophobicity Profiles generated before preprocessing for PAK4 & PAK5**. Hydrophobicity profiles of the sequences of kinases PAK4 and PAK5 generated by substituting the amino acid characters with their respective property value (hydrophobicity values given in table 1). The two sequences are known to be closely similar. These profiles would subsequently be divided in equal segments and the neighborhood around the maximum peak in each segment would be converted to an orthogonal plane using Fast Fourier Transformation.

The sequences of floating point values thus generated is plotted with the position of amino acid as abscissa and its attribute measure as ordinate for each dimension *p*. The attribute is analogous to the amplitude of a time-varying non static signal, and the generated graph to the amplitude profile of the signal. Figure 1 describes the hydrophobicity profile of two closely related kinases *PAK4 *(Swiss-Prot [32] accession no: Q8N4E1) and *PAK5 *(Swiss-Prot accession no: O95547). Thereafter, the profile is segmented in equal segments of fixed length and the local maximum is found in each segment. The width of the segment would matter in the quality of results.

**Figure 2:**
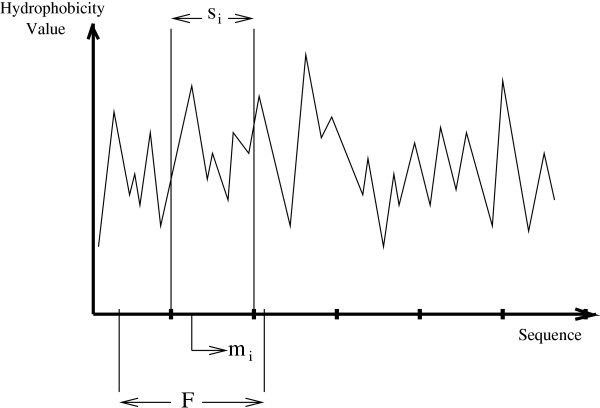
**Preprocessing of Inputs in a single property plane**. The property profile of one of the input sequences in a plane is subjected to segmentation of equal sizes. Maximum peak in each segmented is identified by simple comparison of the heights of the peaks and the a neighborhood of size *F *around the position containing the peak is taken. Each neighborhood is then collectively subjected to fourier transformation. This preprocessing is implemented in each plane of the property profile.

For each dimension *p *pertaining to property *P*_*p*_, let the sequence be divided in *N *equal segments denoted by *s*_*p*,*i *_where *i *∈ {1, 2 ..., *N*} and size of each segment be *S*_*z*_. Also let the positions in each segment where local maximum was found be *m*_*p*,*i *_where *i *∈ {1, 2, ..., *N*}. The maxima is found within the segment in the abcissa by simply comparing the peaks of the property values, as represented in figure 2.

The purport of identifying local maximum *m*_*p*,*i *_in each segment *s*_*p*,*i *_is to do away with bogus peaks in the neighborhood. It is assumed that an amino acid with the highest value of say, *hydrophobicity *would be able to influence the property of the protein the most in the vicinity. It should be noted that a local minima (instead of maxima) in each segment can also be considered for evaluation in the case where a lower value of the property determines the strength. For example, in the property considered here, the minima would mean the highest hydrophilicity. However, it is possible that the local maximum is not able to catch the property in a limited neighborhood, but that aspect is considered in the step that follows.

### Conversion to frequency domain

Around each position *m*_*p*,*i *_a neighborhood of a size *F *is taken and converted to the frequency domain by using Fast Fourier Transformation (FFT) algorithm [33-35]. FFT is faster than other frequency conversion mechanisms and is a linear time algorithm rendering SSS faster [34]. This procedure constraints the value of *F *to a power of 2 (there are other ways with higher time order for fourier transformation that would not put this constraint on the value of *F*). The global alignment during matching is to be done for segments *s*_*p*,*i *_and not for individual amino acids. Positional information of the amino acids within a segment is not available after fourier transformation. Therefore, *F *can be used as a useful manoeuvering parameter while analysis of the alignment output.

The *property profile PP *on segmentation and fourier transformation generates a vector <*v*_*p*,*i *_>. We normalize each segment <*v*_*p*,*i *_> so that their mean is 0 and variance is 1. This procedure is conducted for each dimension *p*. For the two protein sequences to be compared, such two vectors are generated, of say size *n *and *m*.

### Matching

We use *Minimum distance matching *method, a version of the Needleman-Wunsch Algorithm [9]. Let us surmise by considering two lists of vectors <*x*_*p*,1_, *x*_*p*,2_,..., *x*_*p*,*n *_> and <*y*_*p*,1_, *y*_*p*,2_, ..., *y*_*p*,*m *_> respectively. Let *e*_*p*,*i*,*j *_be the mean square distance between *x*_*p*,*i *_and *y*_*p*,*j*_. The mean square distance describes the extent of dissimilarity between the two complex frequency vectors.

Let *M*_*p*,*k *_= {(*x*_*p*,*i*_, *y*_*p*,*j*_)} be defined as a matching of size *k*, pairing *x*_*p*,*i *_with *y*_*p*,*j*_. We need to get the largest matching with the lowest value of dissimilarity. Given the subsets  = {*x*_*p*,1_, *x*_*p*,2_,..., *x*_*p*,*a*_},  = {*y*_*p*,1_, *y*_*p*,2_,..., *y*_*p*,*b*_} and a matching *M*_*p*,*k *_*s.t. *(*k *≤ *a *≤ *n*, *k *≤ *b *≤ *m*), distance between the sets  and  wrt *M*_*p*,*k *_is defined as:



and minimum distance between *X*^*a *^and *Y*^*b *^can be calculated by finding the minimum over *M*_*p*,*k*_. In effect, a penalty of *β*_*p *_is imposed on each non-matching vector, while the dissimilarity measure (msd) is imposed on those which are matching.

**Figure 3:**
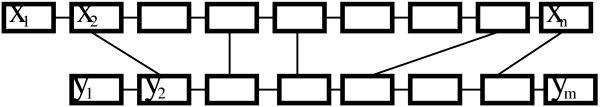
**Matching of segments using dynamic programming**. Matching of the Sequence vectors generated through Dynamic Programming. The method used is a version of the N-W Algorithm. A penalty of *β *is imposed on each non matching of segments while for an accepted match the distance score is increased by the dissimilarity measure between the segments. A matching is defined as an ordered map between the two ordered sets of segments.

**Figure 4:**
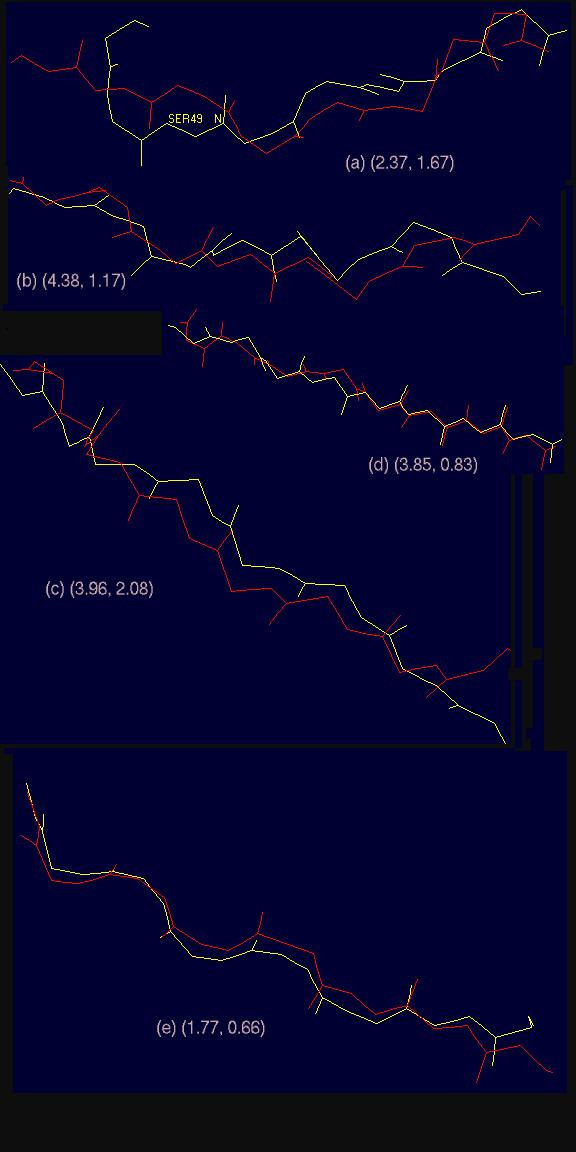
**3D matching for PAKd PAKe using SPDBV magic fit**. 3D images of fit obtained by using SPDBV [30, 31] software's "magic fit" tools. The first value in the bracket is the SSS for the subsequence and second refers to rms value obtained by the tool in ^0^*A*. Color red is used for PKCd and yellow for PKCe. The subsequences in the figures are (a)MKEALSTE & DDSRIGQT (b) ANQPFCAV & QTFLLDPY (c) GKAEFWLD & ANCTIQFE (d) QAKVLMSV & RVYVIIDL (e) RVIQIVLM & RKIELAVF belonging to PKCd and PKCe respectively. All subsequences are completely dissimilar using character based approaches but are found to be similar using SSS. Appreciably low rms values confirms that the subsequences in fig 4a-4e are similar subsequences.

**Figure 5:**
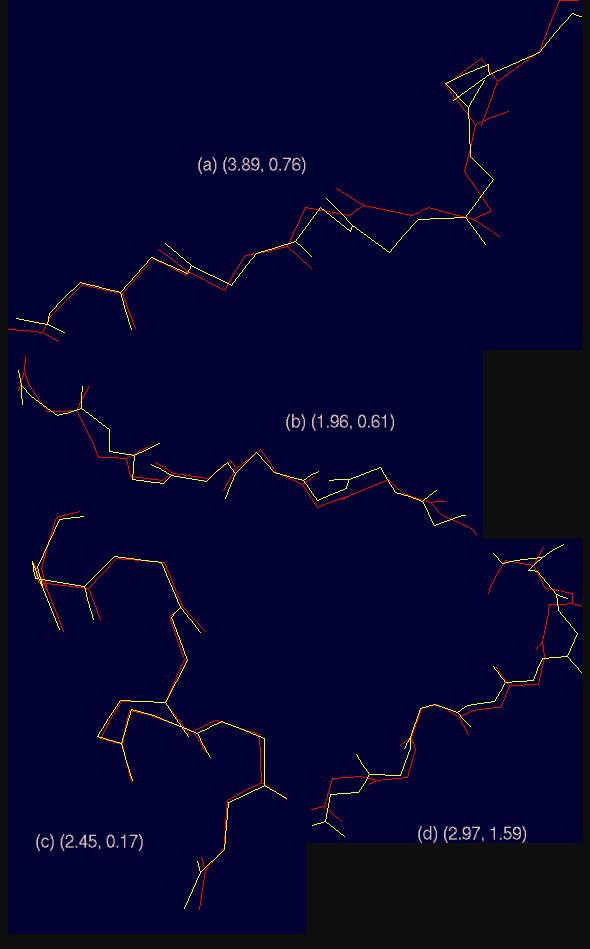
**3D matching for xyna-theau xynz-clotm using SPDBV magic fit**. 3D images of fit obtained by using SPDBV [30, 31] software's "magic fit" tools. The first value in the bracket is the SSS for the subsequence and second refers to rms value obtained by the tool in ^0^A Color red is used for xyna-theau and yellow for xynz-clotm. The two proteins are similar proteins with high BLAST score and overlapping 3D structures. SSS however is still able to catch subsequences that are left as dissimilar by BLAST, and low rms values for captured subsequences confirm the findings. The subsequences in the figures are (a) SCVGITVM & NCNTFVMW (b) GITVWGVA & TFVMWGFT (c) RVKQWRAA & MIKSMKER (d) EDGSLRQT & SGNGLRSS belonging to xyna-theau and xynz-clotm respectively.

**Figure 6:**
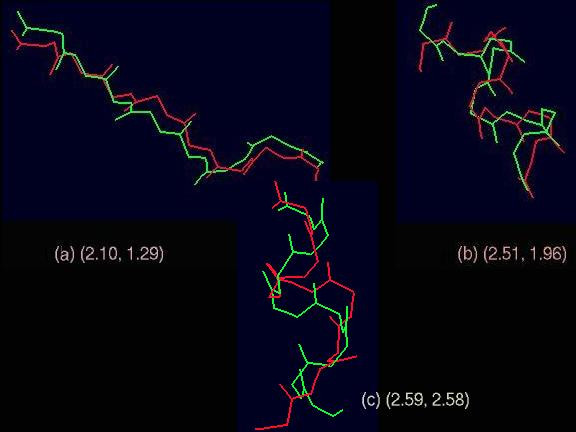
**3D matching for xyna-psefl xynz-clotm using SPDBV magic fit**. 3D images of fit obtained by using SPDBV [30, 31] software's "magic fit" tools. The first value in the bracket is the SSS for the subsequence and second refers to rms value obtained by the tool in ^0^*A*. Color red is used for xyna-psefl and green for xynz-clotm. The subsequences for which structures are shown are (a) NCNTFVMW & RRGGITVW (b) RDSLLAVM & ENGAKTTA (c) YNSILQRE & RQSVFYRQ belonging to xynz-clotm and xyna-psefl respectively. All the subsequences found to be similar are left by traditional algorithms as dissimilar (or unidentical). Interestingly, the subsequences paired up in fig 6b and 6c are not aligned by BLAST but were still found to be similar by SSS and are captured by the same.

The distance measure between the two sequences can be found by using a dynamic programming approach [36] employing a recursive strategy as shown in figure 3.



We determine the optimal matching set *M*_*p*,*k *_which gives the most optimal distance using dynamic programming approach. The optimal matching for all properties is a simple summation of optimal matching for all *p *dimensions. Therefore,  after normalization gives us the Spectral Similarity Score (SSS). Note that the focus of the method is to capture the "interesting" subsequences with similarity in structure, but may not be similar in the alphabetical plane. Hence, this dynamic programming algorithm, which is not the chief concern of the method, can well be replaced suitably by any other matching algorithm for more accurate global alignment.

### Time complexity analysis

The *time order *of an algorithm refered by *O *is defined as the number of operations required as an order of the input size of data. The preprocessing of inputs to replace with attribute amplitudes, and subsequently to identify local maximae in segments is *O*(*n*), while identifying the neighborhood of size *F *takes *O*(*n*) time for *n *residues. FFT takes *Flog*_2_(*F*) time for each vector in the list, and hanning, normalization take *O*(*F*) time for each vector. Since there are *m *= *n/F *vectors in all, it takes m * (*O*(*F*) + *Flog*_2_(*F*)) in all for a sequence. Dynamic Programming requires *O*(*m*^2^) time, if both sequences are assumed to be of equal length. Matching set can also be found in linear time over the number of segments *m*.

If the algorithm is implemented in a database, and queried for fixed values of *F *and segment size, then for a database of size *n *the time required is approximated to *O*(*np*), or linear in time for *p *properties considered at a time.

## Authors' contributions

KG developed the idea into the algorithm, coded the software and tested on examples. Also he interpreted the results and jointly wrote the manuscript. DT fine tuned the parameters of the algorithm and did large scale testing on proteins besides assisting in writing the manuscript. SVV developed perl scripts for automation of testing. KVV supervised the testing of examples, fine tuning of the algorithm and jointly wrote the manuscript. SR assisted in developing the idea and guided the software development and testing.

## Figures and Tables

**Figure 7:**
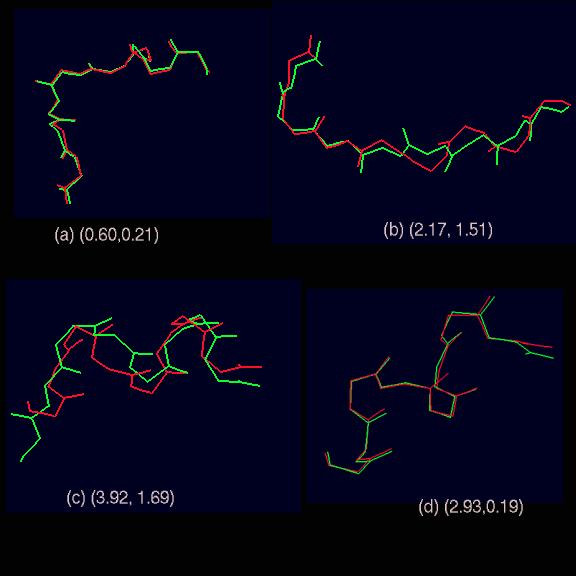
**3D matching for xyna-theau xyna-strli using SPDBV magic fit**. 3D images of fit obtained by using SPDBV [30, 31] software's "magic fit" tools. The first value in the bracket is the SSS for the subsequence and second refers to rms value obtained by the tool in ^0^*A*. Color red is used for xyna-theau and green for xyna-strli. Subsequences for which structures are shown are (a) TTPLLFDG & QTPLLFNN (b) SQTHLSAG & FQSHFNSG (c) VLQALPLL & YNSNFRTT belonging to xyna-theau and xyna-strli respectively. Fig 7a shows a structure refering to matching subsequences that shows that SSS is able to capture subsequences like traditional algorithms also, though it is also capable of picking subsequences like in fig 7c that are not similar on the basis of amino acid characters.

**Table 1:** Estimated Hydrophobic Effect for residual burial. Estimated Hydrophobic Effect for residual burial shown in the second column for each amino acid. These values are substituted for individual amino acid forming a property plane for further preprocessing of inputs in SSS. The values are in kilocalories/mol.

Amino Acid	value [kcal/mol]
Gly	1.18
Ala	2.15
Val	3.38
Ile	3.88
Leu	4.10
Pro	3.10
Cys	1.20
Met	3.43
Phe	3.46
Trp	4.11
Tyr	2.81
His	2.45
Thr	2.25
Ser	1.40
Gln	1.65
Asn	1.05
Glu	1.73
Asp	1.13
Lys	3.05
Arg	2.23

**Table 2:** Distance Matrix for human kinases PAK series. Distance measures *D *between various human kinases. PAK series are closely similar kinases, while PLK1 is a distant relative in the kinome evolutionary tree [27]. Smaller SSS values correspond to strong similarity. GAP (Needleman-Wuntch [9] algorithm implemented in gcg package) scores are in percentage similarity. *F *= 8, *S*_*z *_= 4, *β *= 0.502. It can be seen that the dynamic programming approach used in the SSS algorithm is a simple but effective approach to ascertain global similarity. A replica of the branch of the kinome tree can be generated using the matrix.

	**PLK1**		**PAK4**		**PAK5**		**PAK6**	
	**SSS**	**GAP**	**SSS**	**GAP**	**SSS**	**GAP**	**SSS**	**GAP**

**PLK1**	0.000	100	0.981	39.688	0.976	37.813	0.969	39.264
**PAK4**	0.981	39.688	0.000	100	0.681	69.898	0.845	63.776
**PAK5**	0.976	37.813	0.681	69.898	0.000	100	0.870	58.045
**PAK6**	0.969	39.264	0.845	63.776	0.870	58.045	0.000	100

**Table 3:** Distance Matrix for PKC series in human kinome. Distance matrix for PKC series in Human Kinome. These proteins occur as a distinct branch in the phylogenetic tree of the Human Kinome. GAP results are given as percentages while SSS scores are fractions. Lower SSS scores refer to higher similarity detection. It is seen that SSS with the dynamic programming approach is able to capture phylogenetic relationships between human kinases in the PKC subfamily of proteins. *F *= 16, *S*_*z *_= 8, *β *= 2.5

	**PKCa**		**PKCb**		**PKCd**		**PKCe**	
	**SSS**	**GAP**	**SSS**	**GAP**	**SSS**	**GAP**	**SSS**	**GAP**

**PKCa**	0.000	100	0.4678	85.949	0.7238	61.835	0.7391	63.851
**PKCb**	0.4678	85.949	0.000	100	0.7254	61.029	0.6904	62.944
**PKCd**	0.7238	61.835	0.7254	61.029	0.000	100	0.7149	55.472
**PKCe**	0.7391	63.851	0.6904	62.944	0.7149	55.472	0.000	100
**PKCg**	0.5137	81.081	0.5951	79.464	0.7348	60.589	0.7550	61.695
**PKCh**	0.7160	64.794	0.7371	-	0.7371	53.506	0.6146	76.035
**PKCi**	0.7498	52.072	0.7568	50.357	0.7338	45.098	0.7599	52.909
**PKCt**	0.7113	61.847	0.7474	59.370	0.6068	73.333	0.7451	55.043

**Table 4:** SSS results for PKCd and PKCe kinases. SSS results for the human kinases PKCd and PKCe (BLAST identity score 40%, similarity 57%). Similar subsequences are shown where BLAST is not able to find appreciable similarity with pure character matching strategies. None of the good alignment detected by BLAST were found to be with high SSS scores. Only the sequences with low SSS scores but low BLAST alignments are shown. Smith-Waterman algorithm application SSearch results are also shown. Figures in the last column are created by Magic Fit using the SPDBV software with real pdb files downloaded from the PDB Databank. *F *= 16, *S*_*z *_= 8, *β *= 2.5. PDBids : PKCd = 1BDY, PKCe = 1GMI. The assignments for secondary structure are: *h = helix; b = residue in isolated beta bridge; e = extended beta strand; g = 310 helix; i = pi helix; t = hydrogen bonded turn; s = bend *[37].

	**Seq**	**Segment**	**Subseq**	**msd**	**Blast Result**	**SSearch Results**	**rms**	**Image**
1	PKCd PKCe	(4) [31–39] (6) [46–54]	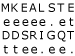	2.31	MKEALSTE DDSRIGQT	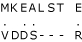	1.67	fig 4a
2	PKCd PKCe	(3) [22–30] (4) [33–41]	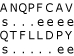	4.38	ANQPFCAV QTFLLDPY	ANQPFCA V QTFLLDP Y	1.17	fig 4b
3	PKCd PKCe	(12) [99–107] (12) [95–103]	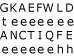	3.96	GKAEFWLD ANCTIQFE	GKAEFWL D ANCTIQF E	2.08	fig 4c
4	PKCd PKCe	(14) [110–118] (15) [111–119]	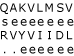	3.85	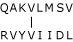	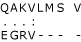	0.83	fig 4d
5	PKCd PKCe	(8) [66–74] (10) [76–84]	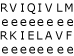	1.77	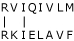	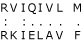	0.66	fig 4e

**Table 5:** SSS results for xyna-theau and xynz-clotm. SSS results for the human kinases xyna-theau and xynz-clotm (BLAST identity score 41%, similarity 59%). Similar subsequences are shown where BLAST is not able to find appreciable similarity with pure character matching strategies. None of the good alignment detected by BLAST were found to be with high SSS scores. Only the sequences with low SSS scores but low BLAST alignments are shown. Figures in the last column are created by Magic Fit using the SPDBV software with real pdb files downloaded from the PDB Databank. *F *= 16, *S*_*z *_= 8, *β *= 2.5. PDBids : xyna-theau = 1GOR, xynz-clotm = 1XYZ. The assignments for secondary structure are: *h = helix; b = residue in isolated beta bridge; e = extended beta strand; g = 310 helix; i = pi helix; t = hydrogen bonded turn; s = bend *[37].

	**Seq**	**Segment**	**Subseq**	**msd**	**Blast Result**	**rms**	**Image**
1	x-theau x-clotm	(32) [259–267] (30) [242–250]	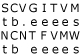	3.89	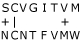	0.76	fig 5a
2	x-theau x-clotm	(36) [288–296] (31) [245–253]	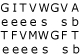	1.96	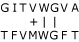	0.61	fig 5b
3	x-theau x-clotm	(27) [215–223] (20) [158–166]	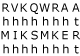	2.45	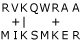	0.17	fig 5c
4	x-theau x-clotm	(20) [160–168] (13) [103–111]	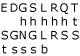	2.97	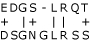	1.59	fig 5d

**Table 6:** SSS results for xyna-psefl and xynz-clotm. SSS results for the F/10 xylanases xyna-psefl and xynz-clotm (BLAST identity score 33%, similarity 52%). Similar subsequences are shown where BLAST is not able to find appreciable similarity with pure character matching strategies. Interestingly the second subsequence does not find alignment in BLAST and is not sequentially similar but produces good alignment. SSearch alignment results are based on Smith-Waterman algorithm. SSearch also did not align the presented subsequences, though it is more sensitive to local and detailed alignments of sequences. Smith-Waterman score was 510 while similarity score in SSearch was 32.984%. Referenced figures show the fit obtained using SPDBV Magic Fit. *F *= 16, *S*_*z *_= 8, *β *= 2.5, *SSS *= 0.783. PDBids : xyna-psefl = 1CLX, xynz-clotm = 1XYZ. The assignments for secondary structure are: *h = helix; b = residue in isolated beta bridge; e = extended beta strand; g = 310 helix; i = pi helix; t = hydrogen bonded turn; s = bend *[37].

	**Seq**	**Segment**	**Subseq**	**msd**	**Blast Result**	**SSearch Results**	**rms**	**Image**
1	x-clotm x-psefl	(37) [297–305] (37) [297–305]	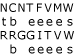	2.10	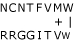	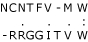	1.29	fig 6a
2	x-clotm x-psefl	(16) [124–132] (23) [184–192]	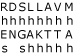	2.51	RDSLLAVMR ENGAKTTAE	DSLLAVM NGAKTTA	1.96	fig 6b
3	x-clotm x-psefl	(07) [054–062] (18) [145–153]	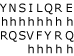	2.59	YNSILQREY RQSVFYRQR	NSILQRE QSVFYRQ	2.58	fig 6c

**Table 7:** SSS results for xyna-theau and xyna-strli. SSS results for the F/10 xyna-theau and xyna-strli (BLAST identity score 47%, similarity 62%). Both the xylanases are exceedingly similar in their structure (*RMS *= 2.13^0^*A *using SPDBV) and therefore close identity in the primary structure is expected as depicted by high BLAST identity score. Similarly, SSearch produces good alignment where character based identity is high. Highly identical subsequences do produce low SSS score (row 1) but non identical subsequences producing low scores are interesting. BLAST does not detect similarity in row 3 subsequences but aligns them. SSearch, however, does not align the two subsequences in row 3. Most of them show similar secondary and tertiary structures. Note the similarity in secondary structures shown below each subsequence. *F *= 16, *S*_*z *_= 8, *β *= 2.5. PDBids: xyna-theau = 1GOR, xyna-strli = 1EOV. The assignments for secondary structure are: *h = helix; b = residue in isolated beta bridge; e = extended beta strand; g = 310 helix; i = pi helix; t = hydrogen bonded turn; s = bend *[37]. *SSS *= 0.731.

	**Seq**	**Segment**	**Subseq**	**msd**	**Blast Result**	**SSearch Results**	**rms**	**Image**
1	x-theau x-strli	(38) [304–312] (41) [329–337]	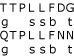	0.60	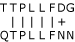	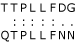	0.21	fig 7a
2	x-theau x-strli	(29) [231–223] (32) [255–263]	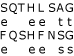	2.17	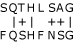	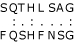	1.51	fig 7b
3	x-theau x-strli	(30) [243–239] (33) [265–273]	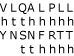	3.92	VLQALPLL YNSNFRTT	VLQALPLL YNSNFRTT	1.69	fig 7c
4	x-theau x-strli	(27) [215–223] (30) [239–247]	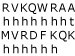	2.93	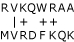	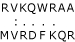	0.19	fig 7d
